# Systems-Biology Approaches to Discover Anti-Viral Effectors of the Human Innate Immune Response

**DOI:** 10.3390/v3071112

**Published:** 2011-07-11

**Authors:** Carsten Münk, Andreas F.R. Sommer, Renate König

**Affiliations:** 1 Clinic for Gastroenterology, Hepatology and Infectiology, Medical Faculty, Heinrich Heine-University, Düsseldorf 40225, Germany; E-Mail: Carsten.Muenk@med.uni-duesseldorf.de; 2 Research Group “Host-Pathogen Interactions”, Paul-Ehrlich-Institut, Langen 63225, Germany; E-Mail: Andreas.Sommer@pei.de; 3 Infectious and Inflammatory Disease Center, Sanford-Burnham Medical Research Institute, La Jolla, CA 92037, USA

**Keywords:** antiviral, innate responses, systems-biology

## Abstract

Virus infections elicit an immediate innate response involving antiviral factors. The activities of some of these factors are, in turn, blocked by viral countermeasures. The ensuing battle between the host and the viruses is crucial for determining whether the virus establishes a foothold and/or induces adaptive immune responses. A comprehensive systems-level understanding of the repertoire of anti-viral effectors in the context of these immediate virus-host responses would provide significant advantages in devising novel strategies to interfere with the initial establishment of infections. Recent efforts to identify cellular factors in a comprehensive and unbiased manner, using genome-wide siRNA screens and other systems biology “omics” methodologies, have revealed several potential anti-viral effectors for viruses like *Human immunodeficiency virus type 1* (HIV-1), *Hepatitis C virus* (HCV), *West Nile virus* (WNV), and influenza virus. This review describes the discovery of novel viral restriction factors and discusses how the integration of different methods in systems biology can be used to more comprehensively identify the intimate interactions of viruses and the cellular innate resistance.

## 1. Introduction

Humans are constantly threatened by a diversity of viruses, and therefore have developed a variety of efficient strategies to fight off infection. Among these strategies, the innate immune system provides a first line of defense against pathogens. Germ-line encoded pattern-recognition receptors (PRRs) recognize pathogens and this exposure rapidly initiates a cascade of events that results in the expression of a variety of genes involved in inflammatory and immune responses [1]. The Interferon (IFN) family of cytokines is recognized as a key innate immune component. Three classes of IFN have been identified (Type I, II and III) that mediate through their cognate receptors the induction of IFN-stimulated genes (ISGs) with antiviral and antimicrobial activities [2]. As a result, viruses have to counter these defenses by incorporating tools that can dampen or overcome the antiviral host defense [3]. The presence of antiviral genes and ISGs is not limited to mammals and found, for example, in fish and birds. Examples for genes instigating an antiviral state are PRRs like the toll-like receptors and RNA helicases [4]. Prominent examples for the discovery of antiviral effector genes are the GTPase Mx1 (myxovirus resistance 1) [5], the protein kinase R (PKR), the 2′,5′-oligoadenylate-synthetase-directed ribonuclease L pathway with the involved proteins OAS and RNaseL and the IFN-stimulated protein of 15 kDa (ISG15) [2]. This review will concentrate on systems-biology methods that can be potentially applied to identify novel antiviral effectors against human viral pathogens like HIV-1, HCV and influenza virus.

## 2. Employing Systems-Biology Approaches for Unbiased Identification of Anti-Viral Effectors

The innate immune system is a complex network of interconnected pathways with multifaceted feedback or feed-forward loops, cross-talk and diverse mechanism of regulation, including post-transcriptional and post-translational modifications [6]. Another dimension, the interplay of the host with various pathogens, adds to this complexity. The host-virus interface is comprised of three fundamental components: (i) recognition and induction of signaling by innate immune receptors, (ii) cellular antiviral responses, and (iii) viral evasion of innate restriction mechanisms. To identify and understand the effective response to viral infections, all three components together are important players that need to be researched to understand the complex host-pathogen relationship. While a candidate gene approach is feasible to begin to dissect the innate immune responses involved in viral infections, that methodology also has multiple limitations and disadvantages. Such analyses restrict the investigation to known components and provide marginal systematic insight into the virus-host circuitry that mediates innate responses to viral infection. Thus, it would be unlikely that such a reductionist approach will elucidate combinatorial effects and emergent properties of molecular systems that ultimately underlie the degree and effectiveness of cellular responses that lead to restriction of infection.

By contrast, systems-level analyses theoretically represent comprehensive and unbiased survey of the host-pathogen interactions that underlie these innate responses. The integration of high-throughput “omics” data such as functional genomics, transcriptomics, proteomics, metabolomics and other approaches that inform us of networks and dynamic system models can provide global insight towards cellular antiviral responses [7,8]. The assimilation of multiple orthogonal datasets can enable the discovery of a global cellular response and regulators of the host-viral relationship. Ultimately, a systems-level analysis will shed light on host susceptibility and resistance to infections, and support the development of novel classes of immune-mediated antivirals, adjuvants, and vaccines.

## 3. Historical Perspective: Discovery of Restriction Factors through Conventional Screening Approaches

Induction of type I interferon regulates the expression of several ISGs that may have direct antiviral properties. A group of proteins with potent anti-viral properties known collectively as “restriction factors” are constitutively expressed or induced by type-1 interferon and are able to limit viral replication by targeting specific steps in the life cycle [9]. In this section, we will summarize the discovery of restriction factors for HIV. These factors were identified with the help of classical genetic screens or by candidate gene approaches coupled with “omics” technologies.

It has been well established that HIV-1 variants lacking certain non-structural genes are limited in their ability to replicate in some cell lines, but show productive infections in other cell lines. In addition, wild type HIV-1 is unable to perform a complete infection cycle in cells of non-Hominidae origin, implicating a species-specific adaptation to viral dependency factors and restriction factors. Conventional systems approaches have expressed cellular cDNA libraries to search for dependency factors like retroviral receptors or to search for restriction factors like TRIM5α (see below). While these methods were quite successful, and identified important proteins they require the knowledge of a previously identified phenotype.

The first restriction factor for HIV-1 was discovered in 2002. It was a well-known phenomenon that the HIV-1 protein Vif is required to counteract a dominant inhibitory factor [10,11]. Cells non-permissive for replication of Δ*vif* HIV-1 were believed to express an antiviral protein that inhibited the infectivity of newly produced particles. Sheehy *et al.* identified this protein, APOBEC3G, by a PCR based cDNA subtraction strategy of non-permissive CEM cells and HIV-1 infected CEM-SS cells. Candidate cDNAs derived from non-permissive cells were expressed in permissive cells and tested for their ability to inhibit viral replication assays [12]. In follow up studies, it was discovered that the cytidine deaminase APOBEC3G is encapsidated in Δ*vif* HIV-1 particles, and during the viral reverse transcription, it can deaminate cytidines in the single stranded DNA. Thus APOBEC3G mutates the viral genomes in target cells and thereby inhibits the replication of HIV [13,14]. The HIV-1 Vif protein counteracts APOBEC3G to circumvent this restriction. Specifically, Vif binds APOBEC3G and recruits an E3 ubiquitin ligase complex that induces polyubiquitination and degradation of APOBEC3G in virus-producing cells [15–17]. In addition, the human genome encodes six more APOBEC3 proteins (APOBEC3A, -B, -C, -D, -F and -H) that can inhibit various retroviruses, endogenous retroelements and DNA viruses [18–22].

The resistance of simian cells to HIV-1 was used to identify another restriction protein that blocks the virus early post-entry at uncoating. Stremlau *et al.* used a retroviral cDNA library of rhesus macaque cells to transduce permissive human HeLa cells and screened for HIV-1 resistant cells [23]. This study revealed that HIV-1 is inhibited by the simian TRIM5α protein, but not by the human orthologue. TRIM5α is constitutively expressed, but interferon treatment can further increase its levels [24]. It is speculated that Rhesus TRIM5α restricts HIV-1 by acceleration of the viral uncoating process that is believed to inhibit the reverse transcription. Inhibition of the proteasome relieves the reverse transcription block in cells expressing rhesus TRIM5α, but interestingly, HIV-1 genomes are still blocked for integration [25]. A very recent study suggests that TRIM5α is a multifunctional component of the innate immune system, and serves not only as a restriction factor (effector), but can also promote innate immune signaling that is triggered by interaction with the retroviral capsid lattice. These data imply that TRIM5α may serve as a pattern-recognition receptor for HIV-1 [26]. Traditionally, viruses are recognized through innate immune sensors as foreign by their viral nucleic acids [27] through cytoplasmic PRRs and viral envelope proteins through TLRs [28]. TRIM5α would be the first described innate sensor recognizing HIV-1 capsid.

Another interferon-induced restriction factor, CD317/BST-2/Tetherin, was independently identified by two groups [29,30]. CD317 is neutralized by the HIV-1 protein Vpu. HIV-1 deficient for Vpu is unable to bud from cells due to ‘tethering’ by CD317 [29]. Vpu interferes with the cell surface expression of CD317 partially by inducing its degradation [31,32]. Neil *et al.* used microarray analysis comparing untreated and IFN-a treated cells to identify this HIV-1 restriction factor. Candidate genes were selected by filtering the results for differential expression and localization of the induced genes [29]. Targeting of Tetherin/BST-2 by Vpu was also elucidated by Van Damme *et al.* [30] based on a quantitative proteomic approach that identified BST-2 as a target for the gamma-herpesvirus immune modulator K5 [33].

## 4. Genomics Technologies Enabled the Discovery of Genome-Wide Host-Pathogen Interactions

Systems-biology approaches that used genome-wide libraries of siRNAs or shRNAs dramatically changed the limitations of the traditionally genetic screens. These experiments were enabled by the development and integration of high-throughput technologies. An important challenge using genome wide siRNA screens is the implementation of rigid methods to filter the enormous amounts of data and identify true hits [34–36]. The detection of potential candidate genes is influenced by many factors like timing and filtering thresholds [35]. Meta-analyses, integrating the data with those previously done in functional or proteomic studies will lead to a list of candidate genes identified independently in multiple studies and will increase the chance of calling a true hit [35,37].

Most genome-wide RNAi screens so far focused on identifying viral dependency factors that facilitate replication rather than identifying innate immune genes involved in restricting viral replication (**Influenza:** [38–40]; **HIV:** [41–43]; **Dengue:** [44]; **HCV**: [45]). However, in addition to identifying host proteins, some genome-wide approaches captured possible innate effectors or restriction factors. In these screens the inhibition of restrictive proteins resulted in an increase of viral replication. For example, the study by Zhou *et al.* [46], who conducted a genome-scale siRNA screen (targeting 19,709 genes) revealed more than 311 host factors important for HIV replication. One gene, GM2A (GM2 ganglioside activator) acted as a potential restricting factor for HIV. siRNAs against GM2A increased HIV replication 2-fold.

Restriction factors against human flaviviruses were identified in two siRNAs screens against *West Nile virus* (WNV) and *Hepatitis C virus* (HCV) [47,48]. Krishnan *et al.* [47] identified 305 genes affecting WNV replication, and of these, 22 genes were found to be potential host resistance factors. IRF3, a critical mediator of several known innate response pathways, was amongst those identified. Its identification suggests that genome-wide genetic perturbations screens and other high-throughput datasets have the potential to reveal novel factors that not only restrict viruses, but also act as effectors of innate signaling cascades. In addition, silencing of the monocarboxylic acid transporter MCT4 (*SLC16A4*) delayed the temporal transition into the replication phase of endocytosed WNV particles [47]. Interestingly, silencing of all 22 genes enhanced both WNV and *Dengue virus 2*, speculating that the innate pathways and effectors might be a shared host resistance strategy against flaviviruses.

Li *et al.* [48] performed a genome-wide screen (targeting 19,470 genes) for HCV-host cell interactions, and found that 262 genes when silenced decreased viral replication. In addition, this study also reported more than 20 factors that may function in anti-viral responses or safeguarding the cells against the stress of infection. The anti-viral mechanisms of these potential HCV restriction factors are not known.

Brass *et al.* [49] discovered 120 dependency factors in their siRNA screen (targeting 17,877 genes) for influenza A virus infection. This study also identified a small number of restricting proteins: PUSL1, TPST1, WDR33, and IFITM1, 2, 3. The interferon-inducible transmembrane proteins (IFITM) conferred basal resistance to influenza virus by blocking an early infection step, but are also inducible by interferons type I and II. IFITM proteins mediated cellular resistance not only to flu, but also to WNV and dengue virus, but not to HCV [49]. Interestingly, Lu *et al.* [50] could show that IFITM proteins are also potent restriction factors of HIV-1 and also inhibit the virus at cell entry. The exact mechanism of restriction is currently not known.

In addition, RNAi studies identified antiviral defense genes in *Drosophila*, their impact on viruses replication in human cells has yet to be elucidated [51].

Certain cellular proteins identified as dependency factors might indeed protect the virus against unknown cellular antiviral proteins, a function that is also achieved by some viral non-structural proteins like the multifunctional NS1 protein of influenza virus. None of the above mentioned studies systematically investigated how non-structural proteins influenced the results of the siRNA screens. In addition, none of the above studies conducted their screens in cells induced to be in an antiviral state. Also, sentinel cells of the immune system would be more appropriate to screen for antiviral effects. Since these cells are not easily transfectible in a high-throughput fashion, one needs to think of alternative cell models representing immune cells. A number of screens mentioned above used non-replicating viruses. Designing screens covering the whole life viral cycle will increase the likelihood of viral PAMPs being recognized and in turn leading to induction of antiviral genes.

## 5. Systems-Based Approach to Detect ISGs as Novel Antiviral Effectors

Numerous gene expression datasets are available to enable a global analysis of the genes involved in the IFN response. Those interferon-stimulated genes are the prime candidates for the discovery of potential novel antiviral effectors, however innate cellular restriction mechanisms may not be limited to the activities of IFNs. Microarray technology (“transcriptomics”) has enabled us to capture the comprehensive picture of changes in the expression profile of cells treated with IFN. The ISG database has cataloged hundreds of upregulated genes after stimulation of human HT1080 cells with IFN-α, IFN-β and IFN-γ [52] and additionally from IFN stimulated human dendritic cells and mouse embryonic fibroblasts (MEFs) [53]. The dataset contains many previously identified host defense ISGs like PRKR, OAS2 and Mx genes. A second database, the INTERFEROME database, is a collection of 43 datasets listing ISGs of various sources and can be analyzed using various computational analysis, including identification of promoter regulatory elements, tissue expression, protein domains and others [54,55] (see list of various databases in Table 1). Another study explored the global transcriptional profiles of different immune cell populations in human peripheral blood mononuclear cells (PBMCs) upon stimulation with type I and II interferons and factors involved in cell-mediated immunity (IL12 and TNFα) and allowed the identification of both cytokine-specific and cell-specific transcriptional patterns [56]. All three datasets and databases represent powerful resources from which to decipher potential anti-viral innate effectors.

In search for anti-HIV ISGs, Barr *et al.* [57] used transcriptional profiling of interferon treated cells. They identified that the TRIM22 protein was able to inhibit HIV-1 particle release, but did not inhibit non-related retroviruses. TRIM22 was shown to target the viral Gag protein by changing its intracellular Gag trafficking. A focused screen on all members of the TRIpartite interaction Motif (TRIM) family of E3 ligases revealed that several family members are restriction factors for HIV and act both at early and late stage of the life cycle [58].

Global expression profiles can also be used to assess the changes of a host in response to specific viral pathogens, *ex vivo* or *in vivo*. For instance, comparative studies have led to gene signatures associated with pathogenic strains [59–61]. Kobasa *et al.* [59] investigated the regulation of the host response to 1918 influenza virus in the bronchi of infected macaques by comparison with a conventional influenza virus and reported a deregulated antiviral response and reduced type-I IFN stimulated genes by the pathogenic 1918 strain. A deregulation in 1918 infected macaques was also reported by Cilloniz *et al.* [60], who describe differential changes in inflammatory and cell death related genes. The study of Billharz *et al.* [61] suggests the 1918 NS1 protein as a contributor to such a deregulated expression profile.

Schoggins *et al.* [62], undertook a global comprehensive approach to screen more than 380 ISGs listed in various databases (e.g., the ISG database) on their effect of viral replication of HCV, yellow fever virus, WNV, chikungunya virus, Venezuelan equine encephalitis virus, HIV-1 and other viruses. They developed a cell-based overexpression assay using a lentiviral cDNA expression system, and subsequently challenged these cells with virus to assess the ability of these ISGs to inhibit replication. They show that different viruses are targeted by unique sets of ISGs, and that some ISGs have additive activity. Interestingly, they reported that several ISGs including ADAR, FAM46C, LY6E and MCOLN2 enhanced the replication of certain viruses, adding another layer of complexity to the system [62]. This study characterized broad acting ISGs that inhibited several different viruses, including proteins such as IRF1, C6orf150, HPSE, RIG-I, MDA5, NAMPT, IRF7 and IFITM3. Other tested ISGs showed only antiviral activity against specific viruses. For instance, genes found to inhibit HCV include DDX60, MOV10, MS4A4A, MAP3K14 and SLC1A1. Some of these genes were only active in cells that responded to interferon (feedback into IFN signaling pathways), other genes seem to act as direct inhibitor of HCV (targeted effector function) as it was shown that a common theme of mechanism of antiviral action is translational inhibition. The regulation of the innate antiviral response can also be regulated through non-coding microRNAs (miRNAs). The report of Pedersen *et al.* [63] was one of the first studies supporting the idea that cellular miRNAs induced through the IFN system can combat viral infection. They showed a differential expression profile of miRNAs upon type I and II IFN stimulation and corresponding antiviral effects. Specifically, downregulation of miR-122, that has been previously shown to be essential for HCV replication, contributes to the antiviral effect of IFN-β [63]. Currently, the innate database (DB) is incorporating information on miRNAs known to regulate an innate immunity-relevant gene, thus providing a more comprehensive picture of immunity [6,64].

New technologies such as next-generation sequencing are opening up new avenues for scientific research by sequencing the total transcriptome including mRNAs, microRNAs and long-coding RNAs. Peng *et al.* [65] performed a comprehensive whole-transcriptome analysis of the host response to SARS-CoV infection and observed a differential expression profile of 500 annotated, long ncRNA. Interestingly, 40% of a subset of these ncRNAs was similarly regulated in response to both influenza virus and interferon treatments suggesting a host response regulated by innate immunity [65].

## 6. Proteomics: The Innate Immune Interactome

A comprehensive identification and understanding of antiviral innate effectors will require monitoring changes not only in the transcriptome or through genetic perturbations of cells with siRNA or cDNA, but also monitoring protein abundance, post-translational modifications and protein-protein interaction networks. One of the first compilations of an innate-immune interaction network occurred with the publication of a manually constructed comprehensive map of toll-like receptor (TLR) signaling network [66]. Additionally, the InnateDB project collates and curates more than 13,000 innate-immune-relevant interactions and enhances pathway-specific networks [67]. This database can identify network “hubs” (*i.e*., highly connected nodes) and “bottlenecks” (*i.e*., key connector proteins central to many paths in the network), which are likely to represent key regulatory nodes in the network

Integrating the innate-immune interactome with the host-viral protein-protein interface would provide a clearer picture of the immediate interconnection of viral components with the host cell innate pathways. Studying the pair-wise interaction landscape between viral protein and host proteins have been undertaken with high-throughput yeast two-hybrid maps. For example, de Chassey *et al.* provide a proteome-wide view of HCV-human protein interactions and discovered that the HCV CORE protein was a major perturbator of the insulin, Jak-STAT and TGFb pathways [68]. Jaeger *et al.* reported a method of purification and characterization of HIV-human protein complexes by an AP-MS approach, that, in future, will be a powerful tool to identify connections between viral proteins, innate effectors, and restriction factors [69].

Interestingly, a recent study focused on the collective global human-pathogen protein-protein interactions (PPI) network of 190 pathogen strains. The authors found that pathogens, both viral and bacterial, tend to interact preferentially with human hub proteins and bottleneck factors in human pathways (proteins with many interacting partners or central to many paths in the network) [70]. Because this meta-analysis used studies applying different methods and goals, some results might reflect a selectivity of the initial studies. However, the analyses indicate that pathogens interact with these central points since they may control cellular processes that are critical for essential steps in pathogen replication like nucleic acid metabolism. Databases harboring host-protein interaction include PIG, the pathogen interaction gateway, that collects host-pathogen PPIs for 206 different pathogen strains [71] and, VirusMint [72,73].

Complementary to genetic perturbation and gene expression screens, studies on proteome changes in cells upon viral infections may provide additional critical understanding of the host-pathogen relationship. Added information can include protein degradation or modification through viral proteins and changes in subcellular localization. Quantitative mass-spectrometry can be used to measure protein abundance, post-translational protein modifications and macromolecular complexes. Several proteomic analyses on the global changes in the proteome after HIV-1 infection have recently been published. *Chan et al.* used liquid chromatography-mass spectrometry coupled with stable isotope labeling and the accurate mass and time tag approach for a quantitative analysis that revealed changes in ubiquitination [74]. Two years later, the same group published a shotgun liquid chromatography-tandem mass spectrometry analysis uncovering two distinct proteomic abundance profiles at two phases in replication [75]. A third group revealed a metabolic rerouting of HIV infected T cells by using two-dimensional differential in-gel electrophoresis proteomic analysis [76].

Global proteomic and metabolomics profiling study has also been used to identify the metabolic interplay occurring during infection with HCV [77]. Integrating computational modeling approaches revealed that mitochondrial fatty acid oxidation enzymes are differentially regulated both in culture and in HCV-infected patients. This study highlights the potential of complementary approaches to elucidate mechanisms by which viruses take over cellular resources for their own replicative advantage.

Proteome-wide analyses, like transcriptomics, do not automatically reveal the factors that act as antiviral effectors. Use of virus mutants, or cells that have defined defects in the innate pathways, can help to identify candidate genes or the direct action of viral proteins on cellular proteins.

## 7. The Advantage of Integrating Systems Approaches

A biological system, like a host-viral relationship, operates through the concerted action of different classes of molecules (DNA, RNA, proteins, metabolites). For each class, “omics” technologies reveal a global view and can be integrated using computational techniques to create underlying networks and reveal over-represented functional classes. Each network represents a specific type of interaction involving different components, such as genes, transcripts, regulatory RNAs, proteins, modified proteins and metabolites. Those networks are highly interconnected and dynamic. For instance, a miRNA can regulate pairs of interacting proteins and in turn, a protein complex might regulate the activity of regulatory RNA. Only the integration of “omics” datasets will lead to a comprehensive view of all processes [78]. For example, a recent study on the reconstruction of a transcriptional network mediating pathogen responses exemplifies the advantages of dynamic integration of datasets. Genome-wide mRNA expression profiling upon pathogen stimuli revealed 125 candidate transcription factors, chromatin modifiers and RNA binding proteins. Silencing of the candidates in presence of the stimulus resulted in a gene signature that enabled the construction of a regulatory network model of 24 core regulators and 76 fine-tuners. This model helped to explain how pathogen-sensing pathways achieve specificity [79]. In addition, the importance of understanding the temporal codes of intra-cellular signaling is becoming increasingly recognized [80].

Similarly, Shapira *et al.* integrated a multi-layered approach to uncover dynamic interactions between H1N1 influenza virus and its human host [81]. They integrated protein-protein interaction data (yeast two-hybrid approach to generate a human protein network interacting with influenza proteins), genome-wide expression profiling, functional genomics and network modeling. Four different strategies were used to delineate the transcriptional response to infection: Infection with wild-type influenza virus; infection with a virus lacking NS1, which is impaired in counteracting the antiviral host response; stimulation with IFNβ and transfecting viral RNA that triggers the RNA-sensing pathway. A comprehensive map of physical and regulatory interactions between influenza and its host was constructed. This led to 1745 selected candidate genes that may play a role in host responses. The functional contribution of these genes to viral replication and IFN production was then tested by siRNA knockdown. This allowed the assignment of specialized roles in the host-pathogen network to each candidate gene product. Many known host responses, (e.g., RIG-I mediated sensing), but also several novel pathways were identified, (e.g., a group of inflammasome-related sensors and a group of proteins that is essential for the control of virus replication (e.g., USHBP1, ZMAT4 and MAGEA11)). This broad and unbiased integration of various datasets in a model of viral-host interactions provides a promising direction for future studies. Although not focused on innate effectors and signaling components, but on host dependency factors, several studies exemplify the integration of multiple datasets to derive sub-networks with over-represented functions that are important for (i) the influenza-host relationship [37,40], or (ii) affecting distinct steps in the life cycle of HIV-1 [42]. In addition, a meta-analysis surveyed several genome-wide datasets to yield a more corroborated set of host cell factors assisting HIV replication. These genes then were used to calculate refined protein clusters specifying cellular subsystems recruited by HIV [35]. Therefore, integration of multiple datasets is effective in discerning cellular networks important for the host-viral interface, and can help define the most attractive targets for the development of novel HIV therapeutics [35].

## 8. Major Challenges in Systems-Biology Research on Host-Viral Interactions

Systems-biology datasets are inherent to (prone to) approach-specific limitations. Factors like experimental assay design, choice of reagents, the existence of false positive and negative activities and furthermore bioinformatics analyses and hit selection contribute to the variance between datasets and the complexity of the final results [82]. Lessons learned from siRNA screening for HIV-1 or Influenza host factors: overlaps between datasets are limited when analyzed at the gene level [82,83], however the concordance is greater at the level of gene function or protein complexes [35,37]. Off-target activities are an inherent problem of large-scale siRNA screening and can be reduced by increasing the confidence in potential hits through secondary validation assays (cDNA rescue experiments), bioinformatic approaches (to identify at least two hit siRNAs targeting the same gene) and integrating several datasets [34,84]. A major factor to consider is the “bias” that is introduced through the necessity of hit selection. While all “omics” approaches start out with an unbiased approach, the choice of criteria used to rank the hits will automatically introduce a bias. The future challenge will be to reduce this bias by integrating several systems-level datasets (as discussed in Section 6), to organize and interpret the generated information and build models that then in turn can be tested experimentally and iteratively refined.

Systems-biology approaches are being employed to complete the picture of the innate pathways sensing viral pathogens and of viral-specific ISGs, however, important questions and answers are still outstanding. (i) A systematic survey of viral proteins or protein-domains counteracting the innate responses. (ii) The impact of variability of different virus strains on the innate immune recognition and antagonizing the cellular effectors (e.g., pathogenic *versus* non-pathogenic strains; pandemic *versus* non-pandemic strains). (iii) The impact of natural variation in the human population. (iv) The discrimination of the innate system between different pathogens and danger signals to mount an appropriate response. The ultimate goal of the systems-based approaches will be to enable the development of therapeutic strategies for antivirals that interfere with viral evasion of host immune defenses. Also, a more global understanding of the innate immune response may lay the groundwork for improving vaccination strategies. Realizing this vision will depend on the formation of broad research consortia sharing their knowledge and resources with the community and combining the wealth of information in intelligent and user-friendly public databases.

## Figures and Tables

**Table 1:** Selected bioinformatics resources for innate immune and host-pathogen research. Note that some descriptions are quoted directly from the website.

	**Database**	**Website Link**	**Details**	**Reference**
**Immune Databases**	ISG Database	http://www.lerner.ccf.org/labs/williams/	A database for Interferon-stimulated genes. Can be queried for functional categories	[52]
	Interferome Database	http://www.interferome.org/	A database for Interferon-regulated genes	[55]
	Innate Database	http://www.innatedb.ca/	Innate immunity-relevant interactions and pathways	[64]
	Reference Database of immune Cells (RefDIC)	http://refdic.rcai.riken.jp/welcome.cgi	Gene expression profiles at the mRNA and protein levels for Immune cells and tissues	[85]
	Innate immune database	http://db.systemsbiology.net/cgibin/GLUE/U54/IIDBHome.cgi	Information on more than 2000 mouse genes related to immune responses	[86]
	PRRDB	http://www.imtech.res.in/raghava/prrdb/	Comprehensive database of pattern-recognition receptors and their ligands	[87]
	Immunome	http://bioinf.uta.fi/immunome	Database for genes and proteins of the human immune system	[88]
	Macrophages.com	http://www.macrophages.com/	Broad repository for data and information about macrophages	-
**Host-Pathogen interactions**	Pathogen interaction gateway	http://molvis.vbi.vt.edu/pig/	User interface for searching available data and tools to predict interactions between host and pathogen	[71]
	Virusmint	http://mint.bio.uniroma2.it/virusmint/Welcome.do	Collection of interactions between human and viral proteins and integration into human protein interaction network.	[72,73]
	HIV-1, Human Protein Interaction Database	http://www.ncbi.nlm.nih.gov/RefSeq/HIVInteractions/	HIV-1, human protein interaction data presented here are based on literature reports	[89–91]
**Protein-Protein interactions**	HPRD	http://www.hprd.org/	Human proteome-wide database for domain architecture, post-translational modifications, interaction networks and disease association	[92]
	MINT	http://mint.bio.uniroma2.it/mint	Interaction database focused on experimentally verified protein-protein-interactions	[73]
	BIND	http://bind.ca	Archive of biomolecular interaction, complex and pathway information.	[93]
	BIOGRID	http://thebiogrid.org/	Database for genetic and protein interaction data from human and model organisms	[94]
	DIP	http://dip.doembi.ucla.edu/dip/Main.cgi	Information on verified protein-protein interactions complied from diverse sources	[95]
	STRING	http://string-db.org/	Databank for known and predicted direct (physical) and indirect (functional) protein associations	[96]
**Pathways**	Reactome	http://www.reactome.org	Literature-curated database of human pathways, which contains 51586 interactions among 1473 human proteins	[97,98]
	KEGG	http://www.genome.jp/kegg	Knowledge base containing genomic, chemical and systemic functional information	[99]
	Pathguide	http://www.pathguide.org/	Collection of biological pathway and molecular interaction related resources	[100]
	PID	http://pid.nci.nih.gov/	Molecular interactions and biological processes in biomolecular pathways	[101]
**Other useful sites**	BioGPS	http://biogps.gnf.org	Free customizable gene annotation portal	[102]
	Symatlas	http://symatlas.gnf.org	Gene expression atlas, integrated into BioGPS Portal	[103]
	Gene Expression atlas	http://www.ebi.ac.uk/gxa/	Curated gene expression archive	-
	ArrayExpress Archive	http://www.ebi.ac.uk/arrayexpress/	Database of functional genomics experiments	[104]
	miRBase	http://www.mirbase.org/	Database of published miRNA sequences and annotation	[105]
	Cytoscape	http://www.cytoscape.org/	Open source software platform for visualizing complex-networks	[106]
